# The importance of having a partner: male help releases females from time limitation during incubation in birds

**DOI:** 10.1186/1742-9994-11-24

**Published:** 2014-03-07

**Authors:** Beata Matysioková, Vladimír Remeš

**Affiliations:** 1 Department of Zoology and Laboratory of Ornithology, Palacký University, 17 listopadu 50, Olomouc 77146, Czech Republic

**Keywords:** Comparative analyses, Food limitation, Latitude, Male incubation, Parental care, Passerines, Paternity, Sexual conflict, Temperature

## Abstract

**Introduction:**

Male contribution to parental care varies widely among avian species. Yet the reasons for this variation, as well as its consequences, are still unclear. Because the amount of care provided by one sex is ultimately constrained by the time available for energy acquisition, contribution by the other sex should increase when overall parental workload is high. We tested this prediction by analyzing male contribution to incubation in 528 populations of 320 species of passerines, where females usually devote more time to incubation than males. Our worldwide sample included species with female-only parental care (the male is not present), incubation feeding (the male feeds the incubating female), and shared incubation (both sexes incubate the eggs).

**Results:**

Overall nest attentiveness was greatest in species with shared incubation followed by species with incubation feeding and lowest in species with female-only care. Nest attentiveness and the degree of male contribution to incubation in species with shared incubation were very strongly correlated, whereas this correlation was absent in females. Interestingly, female contribution decreased towards the equator while male contribution did not change significantly with latitude. Hence, relative male incubation effort increased towards the equator, whereas that of female decreased. In species with incubation feeding, female nest attentiveness increased with the frequency of male feeding.

**Conclusions:**

These findings support the hypothesis that male help is indispensable for increasing nest attentiveness in birds, either in the form of incubation feeding (supply of energy) or direct incubation of eggs. We conclude that energy acquisition constraints might be a potent force driving sex-specific contribution to parental care.

## Introduction

Parental care is widespread and includes a wide array of behaviors, from the simple egg carrying in insects to mouth brooding in fish to elaborate physiological adaptations, e.g. lactation in mammals [1]. It is mostly provided by one sex only (uniparental care) [2]. However, in some species parental care is provided by both sexes. Such biparental care can be found in insects [3], amphibians [4], fishes [5], birds [6,7], and mammals [8]. In biparental species, contribution by each sex varies across species, which is also true for birds [7]. Various ecological and social explanations have been proposed to drive the degree of contribution by the sexes, e.g. parentage, strength of sexual selection or adult sex ratio [9-11]. However, much less attention focused on how the contribution of sexes changes with increasing parental workload.

Since the amount of care provided by one sex is limited by the time available for energy acquisition, contribution by the other sex should increase when overall parental workload is high. This hypothesis was not tested directly yet. Indirect tests were provided by mate removal experiments, where decreasing parental contribution by one partner to zero (i.e. removal) led most often to incomplete compensation by the other partner [12]. However, this experimental design did not differentiate between restraint and constraint, or willingness vs. ability to compensate. The only test across species was conducted in birds, where paternal contribution to care increased with duration of parental care in many, but not all, avian families. However, this test was again indirect as the length of care was taken as a proxy of the overall intensity of care without directly quantifying overall intensity of care [7].

Incubation in birds is an excellent model in which to study limits on parental care and scaling of parental effort. First, it is an extremely time- and energy-consuming behavior [13,14] with a demonstrated trade-off between parental effort and self-maintenance [15]. Moreover, this trade-off can be exacerbated by low food supply common during early breeding season when incubation usually takes place, which further limits the amount of energy an individual can obtain during a foraging bout [16]. Second, both uniparental and biparental incubation can be commonly found in birds, providing necessary variation for comparative analyses. This is also true in songbirds (Passeriformes), where female always incubates (either with or without male help) but species differ a lot in their nest attentiveness, which can range from less than 40% to 100% [17-19].

Males can help incubating females in several ways. First, they can contribute indirectly by feeding females on the nest [20,21]. Second, males can also contribute directly by sharing the incubation with females [22,23]. Due to uncertain parentage, stronger sexual selection on males or higher cost of parental care to males, females usually contribute more to parenting than males [11,24,25], which applies also to incubation [23,26]. However, in some species, females might approach their maximum working capacity allowed by time available for foraging and energy they are able to obtain [27,28]. Under these conditions male participation can be the only way how to increase overall nest attentiveness.

In this study we tested this prediction by analyzing consequences of male contribution to incubation in a large sample of songbirds across the world. We do not claim that greater attentiveness is always better, because species certainly differ in their optima for attentiveness depending on their life history and environment. However, we believe that studying scaling of nest attentiveness with sex-specific parental contribution and comparing different incubation strategies in terms of overall attentiveness can reveal how important male contribution to parental care is in incubating birds. Thus, we studied 1) scaling of total nest attentiveness with both direct and indirect male help. Here we predicted that total nest attentiveness would increase with male help. We also studied 2) patterns of nest attentiveness across species with different forms of male contribution to incubation. Here we tested i) whether nest attentiveness was greater in species where males helped as compared with species with female-only incubation and ii) which of the two modes of male help (direct or indirect) was associated with higher total nest attentiveness. As parents respond evolutionarily to each other’s effort [12], 3) we compared attentiveness of uniparental females with that of biparental females to examine how male help influenced female behavior. Moreover, since latitude is a strong correlate of life-history strategies and hemispheres systematically differ in many life-history and parental care traits [16,29-31], 4) we tested whether male and female contribution varied geographically while controlling for unequal distribution of clades across latitude.

## Results

Altogether we collated data on nest attentiveness in 528 populations of 320 songbird species belonging to 72 families distributed worldwide (Additional file 1: Figure S1), which provided us with extraordinary diversity in behavior to test our hypotheses (Table 1).

**Table 1:** Descriptive characteristics of nest attentiveness (%) in species with different incubation strategies

**Incubation strategy**	**Mean**	**Median**	**Range (min–max)**	**SD**	***N *** **(species)**	***N *** **(populations)**
Female-only care	65.4	68.9	32.7–79.2	11.5	40	51
Female with male incubation feeding	78.2	78.1	51.0–97.8	10.4	156	306
Shared incubation	87.0	90.0	58.2–100	10.5	124	171
Shared incubation (Female contribution)	51.5	52.0	20.9–89.3	13.3	124	171
Shared incubation (Male contribution)	35.5	37.3	3.4–73.6	15.3	124	171

**Figure 1:**
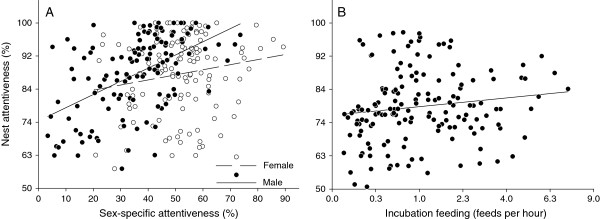
**Relationship between total nest attentiveness (squared) and A) male and female nest attentiveness, and B) incubation feeding (square-root transformed) in species with biparental incubation (*N*** **= 124) and male incubation feeding (*N*** **= 156), respectively.** Points are species averages and fitted lines are ordinary linear regressions for illustrative purposes only.

**Figure 2:**
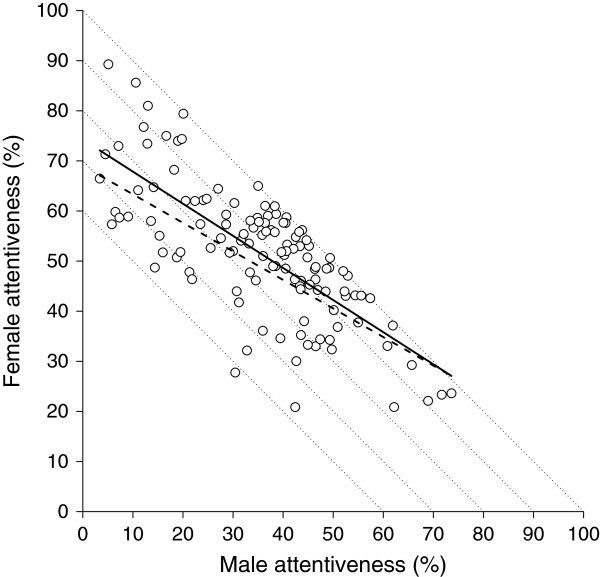
**Relationship between female and male attentiveness in species with biparental incubation (*N*** **= 124).** Full line is ordinary linear regression fit whereas dashed line is phylogenetically corrected fit based on population-level analyses. Dotted lines connect points with identical total nest attentiveness. Inspection of the two regression lines (especially their crossing with dotted lines of equal total attentiveness) suggests that at lower total attentiveness (ca. 70%) female contribution is much higher than male contribution, whereas at higher total attentiveness (ca. 90%) sex-specific contribution is more equal.

To determine whether nest attentiveness increased with male contribution we analyzed the relationship between total nest attentiveness and i) male attentiveness and ii) incubation feeding. In species with shared incubation there was a strong positive correlation between total nest attentiveness and the degree of male contribution (*r* = 0.66, *F*_1,119_ = 91.76, *P* < 0.001; Figure 1A, Additional file 2: Table S1) but not with the degree of female contribution (*r* = 0.12, *F*_1,119_ = 1.85, *P* = 0.263; Figure 1A, Additional file 2: Table S2). This result was confirmed by the observation that female attentiveness decreased with increasing male attentiveness with a slope significantly less than one (slope = −0.57, SE = 0.05, *F*_1,122_ = 112.78, *P* < 0.001; Figure 2, Additional file 2: Table S3). In species with male incubation feeding there was a positive correlation between nest attentiveness and the rate at which the male fed the incubating female (*r* = 0.19, *F*_1,151_ = 5.61, *P* = 0.035; Figure 1B, Additional file 2: Table S4). Incubation feeding rate in these species ranged from 0.02 to 7.33 feeds per hour (mean ± SD = 1.35 ± 1.45, median = 0.81, *N* = 156 species).

**Figure 3:**
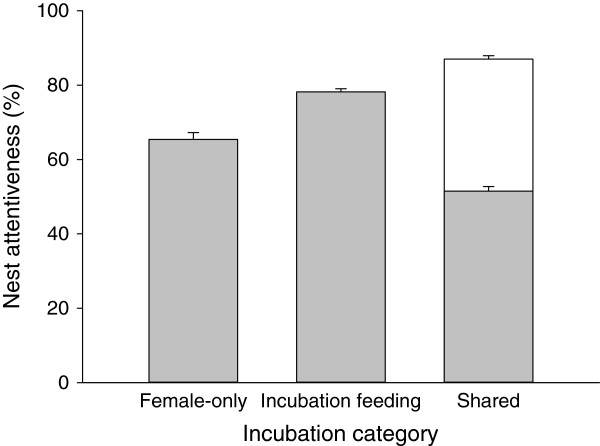
**Total nest attentiveness in species with different incubation strategies.** Female attentiveness is depicted in grey, male attentiveness in white.

**Figure 4:**
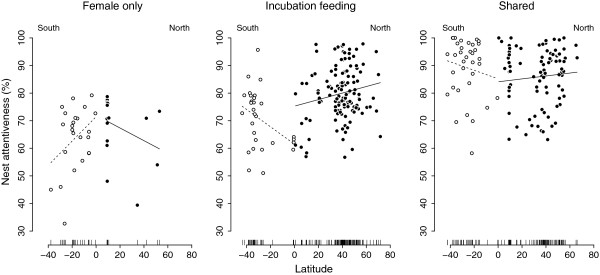
**Total nest attentiveness in relation to latitude (degrees S or N) separately for the three incubation strategies and the two hemispheres (overall *N*** **= 320).** Points are species averages and fitted lines are ordinary linear regressions for illustrative purposes only.

When comparing incubation strategies, total nest attentiveness was lowest in species with female-only care followed by species in which males fed incubating females and highest in species with shared incubation (*F*_2,312_ = 25.30, *P* < 0.001; Figure 3, Additional file 2: Table S5). Moreover, there was also a significant interaction with hemisphere, with greater differences among incubation strategies in the southern hemisphere (*F*_2,312_ = 3.71, *P* = 0.033; Figure 4, Additional file 2: Table S7). Comparing only female nest attentiveness (i.e. using only female attentiveness in species with shared incubation), it was lowest in species with shared incubation, followed by species with female-only care and highest in species with male incubation feeding (*F*_2,314_ = 65.61, *P* < 0.001; Figure 3, Additional file 2: Table S6).

**Figure 5:**
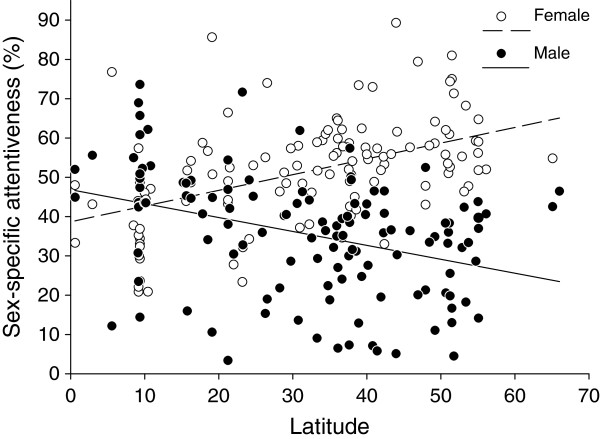
**Relationship between male and female nest attentiveness and geographic latitude (degrees S or N) in species with biparental incubation (*N*** **= 124).** Points are species averages and fitted lines are ordinary linear regressions for illustrative purposes only.

When analyzing geographic variation of female attentiveness in all species, it was lower in the southern hemisphere (*r* = 0.15, *F*_1,314_ = 7.56, *P* = 0.008) and closer to the equator (*r* = 0.23, *F*_1,314_ = 17.55, *P* < 0.001; Additional file 2: Table S8). When analyzing geographic variation of sex-specific attentiveness in species where both sexes incubate, male attentiveness did not change with latitude (*r* = 0.15, *F*_1,120_ = 2.81, *P* = 0.120; Figure 5, Additional file 2: Table S7) whereas female attentiveness was lower in species living closer to the equator (*r* = 0.31, *F*_1,120_ = 12.48, *P* = 0.001; Figure 5, Additional file 2: Table S8).

## Discussion

Patterns of relationships between total attentiveness and male and female contributions suggest that total attentiveness is strongly limited by available time and energy. Total nest attentiveness was positively related to the amount of male contribution to incubation regardless of whether male help was direct or indirect. Namely, it increased with increasing male nest attentiveness, whereas it did not significantly increase with female attentiveness. Similarly, the more often the male fed the incubating female the higher was total nest attentiveness. Furthermore, the male’s presence was revealed to be necessary for high average total nest attentiveness, as it was highest in species in which the male participated in incubation followed by species with male incubation feeding, while it was lowest in species where the male did not help at all. As in other parental care and life-history traits, nest attentiveness varied geographically: female nest attentiveness was lower closer to the equator and in the southern hemisphere.

Our analyses revealed that direct male incubation is a very effective strategy to achieve high total nest attentiveness. We showed that i) average total nest attentiveness was highest in species with shared incubation and ii) in these species total nest attentiveness increased with increasing male attentiveness, whereas it did not significantly change with female nest attentiveness. This is in line with our suggestion that females are prevented from achieving high nest attentiveness by energetic limits and only male help can break these fundamental limits. Of course, females can increase nest attentiveness to a certain extent even when incubating without any male help and in species with female-only care female attentiveness and total attentiveness are the same. However, the low average nest attentiveness in these species as compared to species with male help suggests that there is an upper limit that can be overcome only with male help.

Two caveats are worth mentioning. First, it is important to note that the limitation we identified here is not caused by insurmountable physiological constraints (sensu [32]) but more probably by prohibitive fitness costs of extreme female workload. Consequently, these limits can be modified by species-specific life history, for example foraging behavior or food. This is evidenced by relatively high nest attentiveness in several species with female-only care, e.g. some tropical manakins and cotingas. Second, we do not claim that higher attentiveness is always better. Species have certainly their specific optima given by peculiarities of their life history and environment. However, our results show that high nest attentiveness in songbirds is generally achieved due to extensive male help and we interpret this finding as the evidence for energetic limits on uniparental incubation in birds.

Interestingly, although total nest attentiveness was highest in species with shared incubation, average female attentiveness in these species was 14% points lower than in species in which males do not help at all (Table 1). Why would males take such a large part of the incubation effort on themselves or, alternatively, why would females decrease so much their own incubation effort? One explanation is that in species with shared incubation, sexual conflict arose about the amount of parental effort both sexes would invest [33]. Models predict that when parental duties are shared, sexual conflict can lead to lower parental effort per individual than would be expected if that individual cared alone [34,35]. Our results agree with this prediction. However, the male’s contribution was still on average 16% points lower than the contribution of the female, which is in agreement with a general observation that females usually care more [11,23,24,26].

Another strategy for releasing the incubating female from time and energy limitations, and thus increasing nest attentiveness, is male incubation feeding [20,21]. In species where only females incubate, substantial increase in nest attentiveness is achieved due to male incubation feeding (13% points difference between species with and without incubation feeding; Table 1). Incubation feeding was traditionally believed to have rather symbolic function without any clear energetic benefit for the incubating female [36]. Later experimental studies of single species showed that incubation feeding is an important source of energy to incubating females [37,38]. This view was supported by this study, showing a positive correlation between incubation feeding rate and female nest attentiveness in songbirds from across the world, see also [21].

Average total nest attentiveness was higher in species with male care (both direct and indirect) compared to species with female-only care. However, the two types of male help had different consequences for total nest attentiveness, with higher attentiveness in species with direct male contribution (shared incubation) than in species with indirect one (feeding the incubating female). Why would species adopt different modes of male help? Species-specific life history and environmental factors might dictate which way of male help evolves. For example, in some species territory defense may be more important than higher nest attentiveness. Direct participation in incubation is in conflict with territorial behavior because it is not possible to incubate and defend the territory at the same time [39]. Incubation is also connected with elevated levels of prolactin and low levels of testosterone [40]. Low testosterone level has a negative effect on male territorial behavior [41,42]. Thus, incubation feeding may be a solution of this trade off between the need to help with incubation and to defend the breeding territory. Unfortunately, we know nothing about prolactin levels of males feeding incubating females [43]. Another cost of male incubation might be lost mating opportunities [44] and incubation feeding could be a solution enabling males to simultaneously help the incubating female and obtain some extra-pair matings. These hypotheses remain to be tested in the wild.

We found an interesting geographic pattern in species with shared incubation where female attentiveness decreased towards the equator whereas male attentiveness did not change. Why should males of species living closer to the equator provide relatively more parental care (i.e. in relation to females, see Figure 5)? In tropical birds, the level of extra-pair paternity is considered to be lower compared to species living in temperate regions [45-47] but see [48]. As previously shown, the amount of male parental care during incubation is negatively related to the degree of extra-pair paternity across species [26]. Hence, the higher relative male share of attentiveness in tropical species revealed by our analyses may be the result of higher male willingness to invest into the offspring because of higher certainty of paternity. Male parental care may be also enhanced by low testosterone levels, which is typical for tropical species [49-51]. Biparental care is predicted to be stable when reduced care by one parent is partially compensated by its partner [12,34,35]. Hence, due to higher certainty of paternity males of tropical species may be willing to compensate more, which would consequently allow females to lower their contribution in nest attentiveness. Thus, the pattern we revealed could be a result of an interaction between paternity certainty and sexual conflict over parental care.

Besides paternity and sexual conflict, there are several environmental conditions that might be driving this geographic pattern. First, in songbirds females always incubate during the night. At the same time, nights are longer closer to the equator, which could lead to greater exhaustion of incubating females. Consequently, this would require relatively more help from males during daytime. However, larger clutches and probably colder nights further from the equator, leading to higher metabolic rates [52], should lead to greater female exhaustion there and would predict the opposite. Second, if tropical species are more food-limited, this could lead to more extensive male help with parenting duties. For example, in acrocephaline warblers, males help more in environments with fewer food resources [53]. An exacerbating intrinsic factor could be the generally lower metabolic rate in tropical species, which could cause females to reach their working capacity faster [54]. All these hypotheses are amenable to experimental tests.

In this study we showed that a substantial increase in total parental effort during highly demanding parental behaviors is correlated with increasing male help. Furthermore, we showed that this is true regardless of whether male help is direct or indirect, suggesting a crucial role of male participation in parental duties. Future studies should determine which environmental and social factors favor one or the other of the two alternative male strategies.

## Materials and methods

### Data collection

We collected data for this study from the literature. For data concerning incubation behavior (nest attentiveness and incubation feeding) of songbirds in Europe, North America, Africa, Australia & New Zealand, Central America, and India & Pakistan, we started with respective major compendia, namely *The Birds of Western Palearctic - vols. 5 – 9; Birds of North America; Birds of Africa - vols. 4 – 8; Roberts Birds of Southern Africa; Handbook of Australian, New Zealand, and Antarctic Birds - vols. 5 – 7; Life History of Central American Birds - vols. 1 – 3; Handbook of the Birds of India and Pakistan - vols. 4 – 10*[55-73]*.* We also searched life-history accounts of Neotropical songbirds available online (neotropical.birds.cornell.edu/portal/home). Table A1 [21] was also used to locate articles on incubation feeding (see below).

We used Web of Science (WoS, available since 1945) to find articles that were either published after the above-mentioned compendia had been published, or which were not included in them. We searched by the scientific name of every species in European, North American, Australian, and New Zealand songbirds for all articles whose title or abstract included any of the following words: attentiveness, breeding biology, incubation, nest, nesting or parental care. We discarded all articles that were conducted on birds breeding in captivity and on introduced species. We located additional sources from literature cited in papers obtained in the above-mentioned ways. For practical reasons (too many species with too little information published), we did not search WoS in the same way for birds of Africa, South and Central America, and Asia. We also examined all volumes of available major local zoological journals, many of which are not indexed by WoS. For details see Additional file 3.

We categorized species into three groups based on their incubation behavior. First, we had species with shared incubation where both sexes contribute to warming the eggs. Second, we had species with incubation feeding where the male feeds the incubating female on the nest. In this group we included only species for which we had quantitative information on the rate of incubation feeding. Third, we had species with female-only incubation in which the male does not help during incubation at all. In this group we included only species with female-only care (in which the male is not present at all during offspring care; [6] to be sure that the female incubates without any help from the male (e.g. guarding, vigilance).

From the articles we found, we extracted data on total nest attentiveness (percentage of daytime hours parents spent incubating the eggs). In species with shared incubation, we also extracted data on male and female contribution to nest attentiveness. In several cases the male was either missing or stopped helping the female after being handled by researchers. In these cases we did not count male’s contribution as zero (as we did if the male in a particular pair did not help but was present and his lack of help was not an obvious result of being handled). Instead we excluded data on these particular pairs. This treatment affected eight populations. We also excluded species in which incubation was shared among more than two individuals (*N* = 7). In four species there were both monogamous and polygynous males, and male’s share differed between these male categories. Here we used data for monogamous males only. In all species, we excluded data on incubation during the night, the laying period, and the hatching day.

If there was no information on nest attentiveness in the original article but the lengths of incubation (on) and foraging (off) bouts were provided, we used those numbers to calculate nest attentiveness as: 100 * (mean on-bout duration/(mean on-bout duration + mean off-bout duration)) [74]. The correlation between nest attentiveness obtained directly from the original articles and calculated in this way was very high (r = 0.95, *N* = 383; B. Matysioková, unpubl. data).

We obtained data on male incubation feeding in North American, Australian, and New Zealand songbirds from Table A1 in [21] and supplemented it with studies from other continents that we located during our search. From all articles we extracted the number of feeds per hour by males to females on the nest. We excluded data on off-nest feeding and feeding during other parts of breeding cycle (e.g. egg laying). Incubation feeding can be very rarely observed among species with shared incubation (5 out of 175 species for which we had data on incubation feeding had shared incubation; B. Matysioková, unpubl. data). Hence, in order to avoid confusion, we excluded these five species from our dataset.

In order not to give more weight to a particular breeding pair just because it was sampled more intensively, we decided to use means not weighted by sample size. Hence, we did not directly use data provided in Table A1 in [21], but went to primary literature and recalculated the data on incubation feeding instead. This treatment had almost no effect on final data set since correlation between weighted and unweighted means of incubation feeding per hour in populations from which both estimates were available was very high (r = 0.99, *N* = 44).

From all studies we used in our analyses we also extracted information about the location of the study site. We used this information to record the geographic latitude where the study was conducted using Google Earth. If there were more articles with the same type of data in one species (total nest attentiveness, male and female nest attentiveness, incubation feeding, and latitude), we used averaged values for descriptive purposes (e.g. in Table 1). However, for statistical modeling we used population-level data (see below). If the data on study location was missing we excluded the population from our dataset (*N* = 8). For each species in our dataset we obtained the adult body mass (g) from compendia listed above and from ref. [75]. We could not find data on body mass of *Corvus leucognaphalus* and *Alectrurus risora*. Hence, for data on *C. leucognaphalus,* we used the average of body masses of species in genus *Corvus* of similar size (42–46 cm; [76])*.* For data on *Alectrurus risoria* we used body mass of *A. tricolor*, the only other species belonging to the genus *Alectrurus*.

### Statistical analyses

We used phylogenetic comparative methods to analyze factors explaining incubation behavior in birds. Such methods adjust analyses for the shared evolutionary history of species, which causes nonindependence of data and thus violates assumptions of ordinary statistical approaches [77]. We used phylogenetic generalized least squares using function “gls” in the package “ape” of the R language [78], and simultaneously estimated phylogenetic signal in data using the λ (lambda) parameter [79,80].

Most comparative analyses use only one phylogeny and species averages of traits. However, to account for phylogenetic uncertainty, we used a sample of 100 trees from a recent avian phylogenetic tree ([81]; birdtree.org). We used the Hackett constraint (see [81]), but analyses run on trees with the Ericson constraint generated identical results and are not reported. We excluded four species from the analyses, because they were not included in the phylogenetic trees we used. For many species, we had more than one observation of the response variable. To account for intraspecific variation, we randomly selected one observation for each species for every analysis (i.e., 100 analyses on 100 phylogenetic trees). In this way, we simultaneously accounted for both phylogenetic uncertainty and intraspecific variation. We were not able to use an estimate of intraspecific variation (e.g. SE) directly in the models, because currently implemented methods enable only one predictor in a given analysis (e.g. function “pgls.Ives” of the “phytools” package for R [82].

We fitted six statistical models; their structure and associated hypotheses or questions are detailed in Table 2. Comparative analyses do not test causality and thus it is to a certain extent arbitrary which variable is treated as dependent and which as independent in statistical models. For consistency, we always treated total nest attentiveness as a dependent variable (or female nest attentiveness in Models 3 and 6). However, we were also interested to find out whether male and female contribution to attentiveness changed with latitude. Thus, we fitted two additional models where male and female contribution to attentiveness was dependent variable, respectively. These two models were similar to Model 1 and 2 (Table 2), but enabled us to analyze geographical patterns in sex-specific contribution to attentiveness.

**Table 2:** Structure of fitted models and their associated hypotheses or questions

**Model**	**Response variable**	**Predictors**	**Species**	**Hypothesis or question**
1	Total nest attentiveness	Male attentiveness, body mass, hemisphere, latitude	Shared incubation	Total nest attentiveness increases with the intensity of direct male help
2	Total nest attentiveness	Female attentiveness, body mass, hemisphere, latitude	Shared incubation	Total nest attentiveness and female contribution to attentiveness are not correlated
3	Female attentiveness	Male attentiveness	Shared incubation	Male contribution increases with total nest attentiveness, female contribution does not
4	Total nest attentiveness	Incubation feeding, body mass, hemisphere, latitude	Incubation feeding	Total nest attentiveness increases with the intensity of indirect male help
5	Total nest attentiveness	Incubation category, body mass, hemisphere, latitude	All	Male help (direct or indirect) increases average total nest attentiveness. Do direct vs. indirect male help differ in their effects on average total nest attentiveness?
6	Female nest attentiveness	Incubation category, body mass, hemisphere, latitude	All	Does direct male participation change average female incubation effort?

In models 1 and 2, we fitted an interaction of male or female attentiveness with hemisphere; in model 4, an interaction of incubation feeding with hemisphere. In models 5 and 6, we fitted two-way interactions of incubation category with hemisphere, latitude, and body mass.

Predictors include incubation category (female-only care, incubation feeding, shared incubation), male care (present vs. absent), body mass (g), hemisphere (North vs. South), and absolute latitude (between 0 and 90).

Models 1 and 2 provide the most straightforward test of the hypothesis that total attentiveness should increase with male contribution, but not with female contribution. However, we recognize that in these models, we regress Total nest attentiveness (i.e. M attentiveness + F attentiveness) against one of its components (either M or F attentiveness). To validate our results obtained by these models, we additionally fitted Model 3 where F attentiveness is regressed against M attentiveness. If the slope was 1, F and M attentiveness would compensate perfectly and sex-specific contribution would not change with increasing total nest attentiveness. If the slope was <1, male contribution would increase with increasing total nest attentiveness. If it was >1, the opposite would be true. This model is not so straightforward to interpret as Models 1 and 2, but is statistically less problematic, and thus for the sake of both clarity and statistical rigor, we present results of all these models.

In our models, we initially fitted only interactions of the main factor of interest (always the first predictor in Table 2) with other factors, except that we did not fit interactions of two continuous predictors. Further, we excluded interactions if they were not statistically significant based on *F*-tests. However, we retained all main factors in the models to avoid biases stemming from excessive use of model selection based on *P*-values [83]. We always transformed data to improve the normality of distribution. These transformations are detailed in Additional file 2: Tables S1*–*S8. All tests were two-tailed and the significance value was set at α = 0.05. For continuous predictors, we also present effect sizes expressed as correlation coefficients calculated as *r* = sqrt(*F*/(*F* + df_error_)) [84]. For each model, we obtained 100 values of parameter estimates, their standard errors, and *F*- and *P*-values. In the main text, we present averages of these 100 values. However, in Additional file 2: Tables S1*–*S8 we present also their 95% confidence intervals.

## Competing interests

The authors declare that they have no competing interests.

## Authors’ contributions

BM and VR conceived and designed the study. BM collected data, VR performed the analyses, and BM and VR wrote the paper. Both authors read and approved the final version of the manuscript.

## Supplementary Material

Additional file 1: Figure S1 Distribution of individual studies across the world. Click here for file

Additional file 2: Tables S1 Tables S2–S8. Full results of PGLS models. Click here for file

Additional file 3 List of journals in which data on incubation behavior was searched for. Click here for file
