# Supplementary material for: The importance of having a partner: male help releases females from time limitation during incubation in birds
Source: Front Zool. 2014 Mar 7;11:24. doi: 10.1186/1742-9994-11-24 (PMC4007620; doi:10.1186/1742-9994-11-24)

Figure S1: Distribution of individual studies in space. Please note that some places harbor (much) more studies than apparent due to the overlap of points. This picture thus serves to identify broad geographic patterns only.

Female only

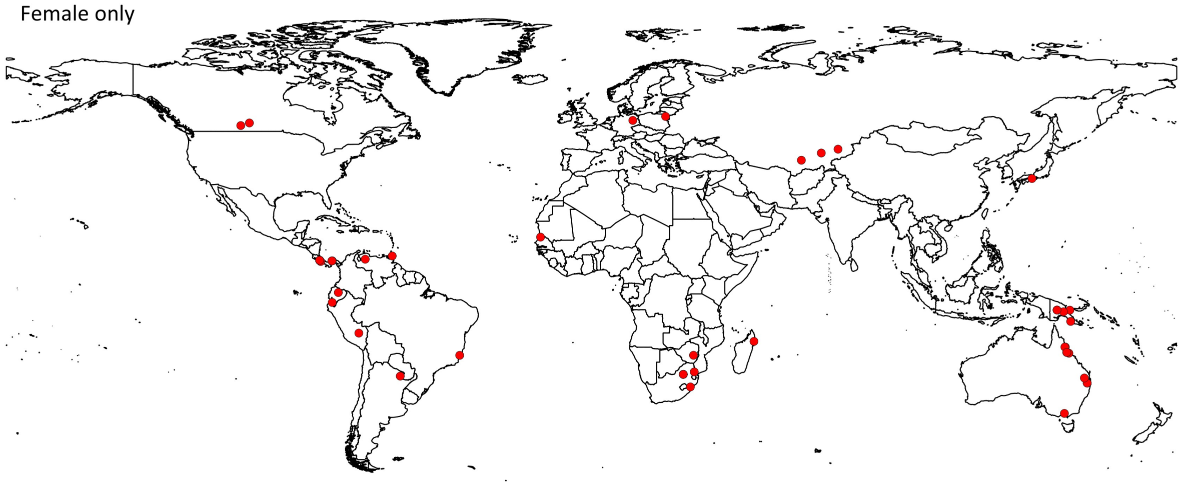

Incubation feeding

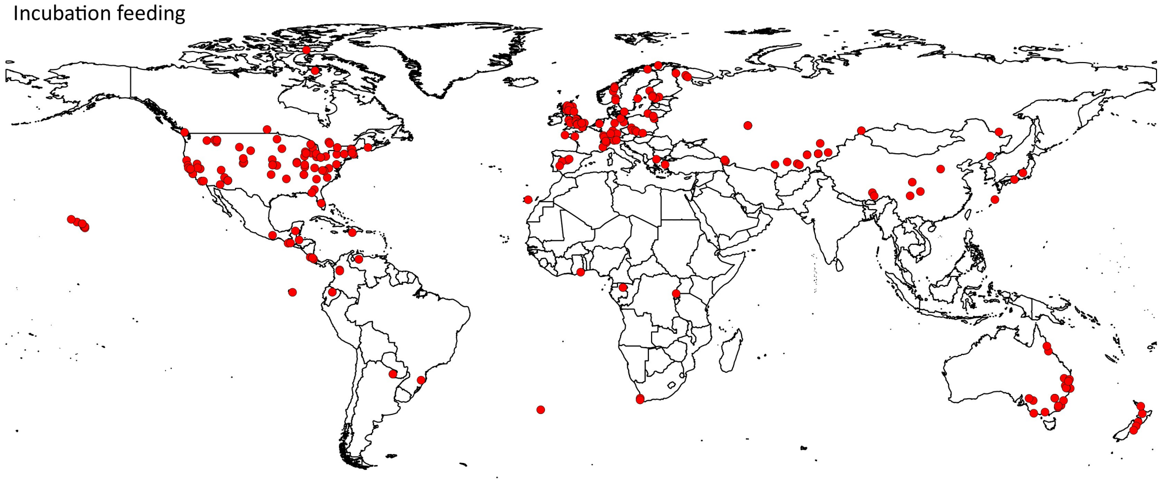

Shared incubation

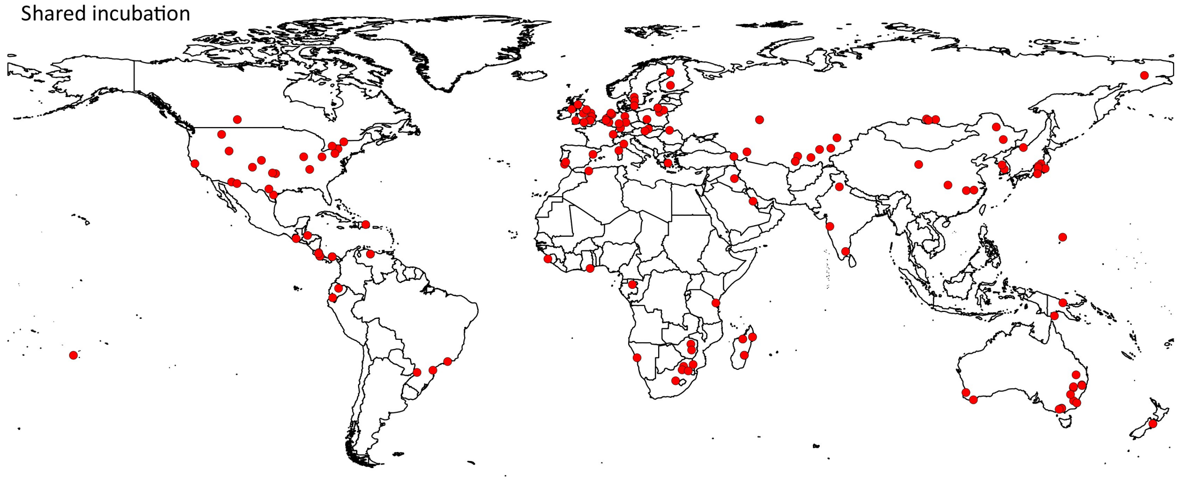

Supplement: Additional file 1: Figure S1 — Distribution of individual studies across the world. [file 1742-9994-11-24-S1.pdf]
