# Supplementary material for: The importance of having a partner: male help releases females from time limitation during incubation in birds
Source: Front Zool. 2014 Mar 7;11:24. doi: 10.1186/1742-9994-11-24 (PMC4007620; doi:10.1186/1742-9994-11-24)
Supplement: Additional file 2: Tables S1 — Tables S2–S8. Full results of PGLS models. [file 1742-9994-11-24-S2.doc]

**Tables S1–S8** presenting full results of PGLS models run across 100 phylogenetic trees and using 100 data sets resampled from population-level data. For every estimate (regression coefficient, SE, *F*- and *P*-value), we provide its mean and 95% confidence interval (LCI–UCI). The response variable and the main predictor of interest are in bold. Reference categories are as follows: “North” for Hemisphere and “Female-only care” for Incubation category. Residual DFs are as follows for models S1–S8: 119, 119, 122, 151, 312, 314, 120, 120, respectively. Factor DFs are always 1, except in Incub. category = 2. The λ values for tables S1–S8 were as follows (LCI–UCI): 0.36 (0.22–0.50), 0.18 (0.04–0.37), 0.33 (0.24–0.42), 0.69 (0.58–0.81), 0.48 (0.40–0.59), 0.61 (0.50–0.73), 0.40 (0.28–0.54), 0.29 (0.11–0.47), respectively. In models S1–S2 and S4-S6, the response variable was squared.

| **S1. Response: Attentiveness** | Estimate |  |  | SE |  |  | F |  |  | p |  |  |
| --- | --- | --- | --- | --- | --- | --- | --- | --- | --- | --- | --- | --- |
| Predictors | Mean | LCI | UCI | Mean | LCI | UCI | Mean | LCI | UCI | Mean | LCI | UCI |
| Intercept | 1818.23 | 1298.94 | 2460.99 | 880.20 | 801.78 | 954.38 |  |  |  |  |  |  |
| log(Body.mass) | 533.47 | 455.16 | 603.77 | 174.19 | 161.82 | 185.42 | 9.51 | 6.43 | 12.87 | 0.004 | 0.000 | 0.013 |
| Latitude | 34.11 | 28.08 | 41.09 | 9.75 | 9.02 | 10.36 | 12.44 | 7.76 | 18.34 | 0.001 | 0.000 | 0.006 |
| Hemisphere (South) | -62.82 | -201.97 | 60.77 | 315.80 | 294.31 | 338.90 | 0.10 | 0.00 | 0.43 | 0.806 | 0.514 | 0.998 |
| **Male share** | 80.58 | 74.97 | 86.46 | 8.44 | 7.79 | 8.94 | 91.76 | 71.83 | 114.75 | 0.000 | 0.000 | 0.000 |

| **S2. Response: Attentiveness** | Estimate |  |  | SE |  |  | F |  |  | p |  |  |
| --- | --- | --- | --- | --- | --- | --- | --- | --- | --- | --- | --- | --- |
| Predictors | Mean | LCI | UCI | Mean | LCI | UCI | Mean | LCI | UCI | Mean | LCI | UCI |
| Intercept | 5679.43 | 5083.92 | 6139.75 | 951.18 | 846.58 | 1072.99 |  |  |  |  |  |  |
| log(Body.mass) | 198.97 | 87.20 | 316.38 | 224.22 | 210.75 | 238.60 | 0.87 | 0.15 | 2.00 | 0.394 | 0.160 | 0.698 |
| Latitude | 11.04 | 2.58 | 20.88 | 12.89 | 12.07 | 13.75 | 0.84 | 0.04 | 2.41 | 0.421 | 0.123 | 0.837 |
| Hemisphere (South) | 490.56 | 277.78 | 783.30 | 394.40 | 369.35 | 420.09 | 1.69 | 0.48 | 4.13 | 0.240 | 0.044 | 0.490 |
| **Female share** | 18.18 | 5.13 | 30.53 | 14.53 | 13.62 | 15.40 | 1.85 | 0.11 | 4.64 | 0.263 | 0.033 | 0.736 |

| **S3. Response: Female share** | Estimate |  |  | SE |  |  | F |  |  | p |  |  |
| --- | --- | --- | --- | --- | --- | --- | --- | --- | --- | --- | --- | --- |
| Predictors | Mean | LCI | UCI | Mean | LCI | UCI | Mean | LCI | UCI | Mean | LCI | UCI |
| Intercept | 69.04 | 66.89 | 71.29 | 3.47 | 3.08 | 4.02 |  |  |  |  |  |  |
| **Male share** | -0.57 | -0.61 | -0.53 | 0.05 | 0.05 | 0.06 | 112.78 | 87.39 | 133.93 | 0.000 | 0.000 | 0.000 |

| **S4. Response: Attentiveness** | Estimate |  |  | SE |  |  | F |  |  | p |  |  |
| --- | --- | --- | --- | --- | --- | --- | --- | --- | --- | --- | --- | --- |
| Predictors | Mean | LCI | UCI | Mean | LCI | UCI | Mean | LCI | UCI | Mean | LCI | UCI |
| Intercept | 4261.97 | 3799.87 | 4837.50 | 984.93 | 896.32 | 1085.06 |  |  |  |  |  |  |
| log(Body.mass) | 90.78 | -55.46 | 230.33 | 173.66 | 162.90 | 183.19 | 0.46 | 0.00 | 1.77 | 0.593 | 0.185 | 0.966 |
| Latitude | 23.46 | 18.24 | 29.87 | 7.75 | 7.32 | 8.28 | 9.37 | 4.97 | 14.53 | 0.006 | 0.000 | 0.027 |
| Hemisphere (South) | -618.75 | -809.80 | -446.15 | 376.42 | 358.72 | 398.01 | 2.79 | 1.40 | 4.65 | 0.115 | 0.033 | 0.239 |
| **sqrt(Incub. feeding)** | 483.02 | 322.10 | 646.08 | 207.85 | 197.48 | 220.27 | 5.61 | 2.26 | 9.25 | 0.035 | 0.003 | 0.135 |

| **S5. Response: Attentiveness** | Estimate |  |  | SE |  |  | F |  |  | p |  |  |  |
| --- | --- | --- | --- | --- | --- | --- | --- | --- | --- | --- | --- | --- | --- |
| Predictors | Mean | LCI | UCI | Mean | LCI | UCI | Mean | LCI | UCI | Mean | LCI | UCI |  |
| (Intercept) | 3422.79 | 3037.06 | 3876.19 | 826.37 | 772.31 | 890.23 |  |  |  |  |  |  |  |
| log(Body.mass) | 216.49 | 141.46 | 281.90 | 127.31 | 122.73 | 132.84 | 2.98 | 1.26 | 5.06 | 0.103 | 0.026 | 0.265 | log(Body.mass) |
| Latitude | 22.09 | 18.62 | 25.64 | 6.64 | 6.40 | 6.95 | 11.17 | 7.70 | 14.60 | 0.001 | 0.000 | 0.006 | Latitude |
| Hemisphere (South) | -942.95 | -1311.95 | -566.13 | 570.04 | 542.22 | 595.02 | 2.86 | 1.05 | 5.27 | 0.120 | 0.022 | 0.307 | Hemisphere |
| **Incub. category (Incub. feeding)** | 1238.99 | 851.65 | 1706.93 | 506.25 | 485.95 | 525.69 | 25.30 | 20.49 | 30.49 | 0.000 | 0.000 | 0.000 | **Incub. category** |
| **Incub. category (Share)** | 2831.53 | 2435.05 | 3261.13 | 496.10 | 475.44 | 515.29 |  |  |  |  |  |  |  |
| Hemisphere (South):Incub. category (Incub.feeding) | 52.52 | -432.92 | 532.27 | 674.10 | 648.12 | 700.59 | 3.71 | 2.23 | 5.28 | 0.033 | 0.006 | 0.109 | Incub.categ*Hemis |
| Hemisphere (South):Incub. category (Share) | 1264.98 | 874.05 | 1737.03 | 659.85 | 636.57 | 687.34 |  |  |  |  |  |  |  |

| **S6. Response: Female attentiveness** | Estimate |  |  | SE |  |  | F |  |  | p |  |  |  |
| --- | --- | --- | --- | --- | --- | --- | --- | --- | --- | --- | --- | --- | --- |
| Predictors | Mean | LCI | UCI | Mean | LCI | UCI | Mean | LCI | UCI | Mean | LCI | UCI |  |
| (Intercept) | 2727.75 | 2379.24 | 3144.71 | 754.22 | 688.12 | 842.12 |  |  |  |  |  |  |  |
| log(Body.mass) | 264.35 | 185.57 | 331.00 | 113.30 | 107.03 | 118.95 | 5.63 | 2.63 | 9.19 | 0.030 | 0.003 | 0.106 | log(Body.mass) |
| Latitude | 23.61 | 20.27 | 27.56 | 5.67 | 5.43 | 5.93 | 17.55 | 12.42 | 24.32 | 0.000 | 0.000 | 0.000 | Latitude |
| Hemisphere (South) | -614.17 | -697.48 | -521.30 | 224.20 | 213.67 | 232.63 | 7.56 | 5.47 | 9.94 | 0.008 | 0.002 | 0.020 | Hemisphere |
| **Incub. category (Incub. feeding)** | 1226.39 | 936.95 | 1597.00 | 350.63 | 333.94 | 366.65 | 65.61 | 53.98 | 80.83 | 0.000 | 0.000 | 0.000 | **Incub. category** |
| **Incub. category (Share)** | -1402.78 | -1679.06 | -1045.39 | 344.41 | 328.91 | 358.19 |  |  |  |  |  |  |  |

| **S7. Response: Male share** | Estimate |  |  | SE |  |  | F |  |  | p |  |  |
| --- | --- | --- | --- | --- | --- | --- | --- | --- | --- | --- | --- | --- |
| Predictors | Mean | LCI | UCI | Mean | LCI | UCI | Mean | LCI | UCI | Mean | LCI | UCI |
| Intercept | 52.09 | 48.89 | 54.83 | 7.84 | 7.04 | 8.78 |  |  |  |  |  |  |
| log(Body.mass) | -3.11 | -3.81 | -2.41 | 1.81 | 1.71 | 1.92 | 3.00 | 1.67 | 4.58 | 0.095 | 0.034 | 0.198 |
| **Latitude** | -0.17 | -0.24 | -0.12 | 0.10 | 0.10 | 0.11 | 2.81 | 1.22 | 5.85 | 0.120 | 0.017 | 0.272 |
| Hemisphere (South) | 5.57 | 4.62 | 6.72 | 3.32 | 3.17 | 3.46 | 2.85 | 1.92 | 3.99 | 0.100 | 0.048 | 0.169 |

| **S8. Response: Female share** | Estimate |  |  | SE |  |  | F |  |  | p |  |  |
| --- | --- | --- | --- | --- | --- | --- | --- | --- | --- | --- | --- | --- |
| Predictors | Mean | LCI | UCI | Mean | LCI | UCI | Mean | LCI | UCI | Mean | LCI | UCI |
| Intercept | 24.47 | 21.83 | 28.08 | 6.50 | 5.93 | 7.24 |  |  |  |  |  |  |
| log(Body.mass) | 4.87 | 4.25 | 5.48 | 1.45 | 1.35 | 1.59 | 11.45 | 7.53 | 15.43 | 0.002 | 0.000 | 0.007 |
| **Latitude** | 0.28 | 0.24 | 0.33 | 0.08 | 0.08 | 0.09 | 12.48 | 7.94 | 16.82 | 0.001 | 0.000 | 0.006 |
| Hemisphere (South) | -3.17 | -4.19 | -2.08 | 2.61 | 2.46 | 2.82 | 1.52 | 0.60 | 2.59 | 0.237 | 0.110 | 0.441 |
