# Supplementary material for: The importance of having a partner: male help releases females from time limitation during incubation in birds
Source: Front Zool. 2014 Mar 7;11:24. doi: 10.1186/1742-9994-11-24 (PMC4007620; doi:10.1186/1742-9994-11-24)
Supplement: Additional file 3 — List of journals in which data on incubation behavior was searched for. [file 1742-9994-11-24-S3.pdf]

S1: List of journals (years or volumes) in which data on incubation behavior was searched for.

Acrocephalus (1989–2011); Acta Biologica Plateau Sinica (vol. 1–12, 14–15); Acta Ornithologica (1933–2011); Acta Ornithologica Lituanica (1989–1994); Acta Zoologica Sinica/Current Zoology (1999–2012); Airo (1990–1999); Apus (1969–2012); Ararajuba/Revista Brasileira de Ornitologia (1990–2011); Australian Birdwatcher/ Australian Field Ornithologist (1959–2009); Aves Ichnusae (1998–2009); Avian Biology Research (2008–2012); Avian Ecology and Behaviour (1998–2011); Avian Science (2001–2003); Bellbird (2006–2010); Berkut (1992–2011); Biotemas (1988–2012); Bird Conservation International (1991–2012); Bird Numbers (1998–2008); Bird Research (2005–2012); Bliki (1983–2009); Bulletin de l'Institut Scientifique, Rabat, section Sciences de la Vie (2003–2012); Bulletin NOS/Malimbus (1964–2006); Bulletin of the British Ornithologists' Club (1940–2012); Bulletín del Grup Català d'Anellament (vol 1–18); Canberra Bird Notes (1968– 2011); Ciconia (vol 1–6, 12, 14); Connecticut Warbler (1999–2010); Corella (1977–2012); Emu (1901–2012); Endemic Species Research/Taiwan Journal of Biodiversity (2000–2010); Fauna Norvegica (1979–1998); Forktail (1986–2009); Great Basin Journal (1998–2004); Hirundo (1988–2010); Honeyguide (1968–2009, except 2001, 2002_vol.48 and 2005_vol.51); Chinese Birds (2010–2012); Chinese Journal of Ecology (1982–2012); Chinese Journal of Zoology (1957–2012); Ibis (1954–1998); Indian Birds (2005–2012); Irish Birds (1977–2012); Journal of Bombay Nat. Hist. Soc. (1886–1900, 1920–2003, 2011); Jack Pine Warbler (1939–1989); Journal of the National Park of Taiwan (vol 12–22); Journal of the Yamashina Institute for Ornithology (1952–2009); Kukila (1985–2012); Living Bird (1962–1981); Mirafra (1984–1989); Mordovskij Ornitologicheskij Vestnik (1998–2003); Murrelet (1980–1988); Muruk (1990–2009); Nature in Singapore (2008–2012); Nebraska Bird Review (1938–2001); Newsletter for Ornithologists /Indian Birds (2004–2011); Nos Oiseau (1949–2009); Notatki Ornitologiczne/Ornis Polonica (1998–2012); Notornis (1943–2012); Open Ornithological Journal (2008–2012); Ornis Hungarica (1991–2009); Ornis Mongolica (2012); Ornitología Neotropical (1990–2008); Ornithological News of Kazakhstan and Middle Asia (2012); Ornithological Observations (2010–2013); Ornithological Science (2002–2012); Ornithologischer Anzeiger (2006–2009); Ornitologiya (1959–1987); Ostrich (1968–2002,2005–2011); Pavo (1963–1997); Pacific Coast Avifauna (1990–1974); Panurus (1989–2012); Phelsuma (1993–2012); Ptaki Śląska (1991–2011); Revista Catalana d'Ornitologia (vol 19–28); Roczniki Akademii Rolnicze w Poznaniu - Ornitologia Stosowana (1966–1995); Safring News (2007–2013); Scientific Journal of zoology (2012–2013); Sichuan Journal of Zoology (1985–2012); Siyoth (2006– 2011); South Australian Ornithologist (1914–2010); Strix (1983–2001); Studies in Avian Biology (1937– 2011); Sylvia (1936–2012); Texas Ornithological Society Bulletin (1967– 2011); The Annals of Oradea University - Biology Fascicle (2005–2012); The Oriole (1948–1988); Tichodroma (1–10, 18–21); TORI/Japanese Journal of Ornithology (1915–2011); Uragus (1926–1929); Vestnik Zoologii (2000–2011); Welsh Birds (1995 –2012); Wilson Journal of Ornithology (2004–2012); Zoological Studies (1996–2012); Zprávy MOS (1976–2010); Казахстанский орнитологический бюллетень (2002–2008).
